# Effect of Acute Grape Seed Extract Supplementation on Heart Rate Recovery in Young Individuals

**DOI:** 10.3390/jcdd12100387

**Published:** 2025-10-01

**Authors:** Dae Sik Song, William Boyer, Trevor Gillum, Sean Sullivan, Iltark Yoon, Junbei Bai, Seung-Jae Kim, Jong-Kyung Kim

**Affiliations:** 1 Department of Kinesiology, California Baptist University, 8432 Magnolia Avenue, Riverside, CA 92504, USA; dsong@udel.edu (D.S.S.); wboyer@calbaptist.edu (W.B.); tgillum@calbaptist.edu (T.G.); ssullivan@calbaptist.edu (S.S.); iltark.yoon@calbaptist.edu (I.Y.); 2 Department of Competitive Sports, Beijing Research Institute of Sports Science, 4 Guangcai North Road, Fengtai District, Beijing 100075, China; junbeibai@hotmail.com; 3 College of Engineering, California Baptist University, 8432 Magnolia Avenue, Riverside, CA 92504, USA; sjkim@calbaptist.edu

**Keywords:** grape seed extract, post-exercise heart rate recovery, grape seed extract, autonomic nervous system, heart rate recovery kinetics

## Abstract

Evidence has suggested that post-exercise heart rate recovery (PHRR) is a useful tool in evaluating cardiac autonomic function. Altered cardiac autonomic function is characterized by heightened sympathetic activation and the abnormal reactivation of the parasympathetic nervous system and is associated with delayed HRR. Although grape seed extract (GSE) supplementation has been shown to increase nitric oxide production and modify sympathetic output, there is limited evidence on its potential beneficial effects on PHRR. We investigated the effect of GSE supplementation on PHRR during sympathetic overactivation induced by muscle metaboreflex activation (MMA) in young individuals. Participants were randomly assigned, via a double-blind, cross-over design, to either receive GSE (300 mg, two capsules) or PL (300 mg, two capsules), with a washout period of at least 72 h. between trials. A submaximal exercise test was performed using a cycle ergometer combined with an isometric handgrip exercise using a handgrip dynamometer and blood flow occlusion by placing a cuff over the brachial artery of the dominant arm. PHRR was measured at 5 s. intervals throughout the experiment. The PHRR was evaluated between GSE and PL at every min. for 300 s. PHRR kinetics significantly improved following GSE supplementation (74.3 ± 7.5 s) compared with the PL condition (86.2 ± 10.4 s). Our results suggest that GSE is effective in improving HRR kinetics during heightened sympathetic activity induced by MMA in young individuals (*p* = 0.034; ES = 0.4). Thus, regular treatment with GSE may provide a nonpharmacological intervention to reduce sympathetic hyperactivity in conditions where excessive sympathetic activity is consistently present.

## 1. Introduction

According to a report from the American Heart Association, 127.9 million adults (48.6%) have one or more forms of cardiovascular disease (CVD), such as coronary heart disease, stroke, hypertension, and congestive heart failure, making it a leading cause of death for both men and women [1]. Evidence indicates that cardiac autonomic dysfunction commonly contributes to the development and progression of CVD [2,3]. Altered autonomic function is attributed to decreased parasympathetic and heightened sympathetic activity. This hypothesis was demonstrated by a previous finding that reducing sympathetic stimulation to the heart through beta-adrenergic blockade positively impacted the disease’s progression [4].

Various procedures to evaluate autonomic function using heart rate variability, cold pressor testing, isometric handgrip testing, and baroreflex sensitivity testing have gained great interest [5]. The exercise test is a simple non-invasive tool to diagnose individuals suspected of having CVD. Post-exercise heart rate recovery (PHRR) is a useful method to assess cardiac autonomic function. PHRR is measured as the difference in maximal HR and HR of passive recovery (i.e., sitting position) every minute immediately after the end of maximal exercise [6,7]. Studies suggest that delayed HRR is associated with impaired parasympathetic reactivation and sympathetic overactivation [8,9]. It has been known that HR obtained at one minute of recovery represents parasympathetic reactivation, whereas HR measured over one minute of recovery represents combined parasympathetic reactivation and sympathetic withdrawal [10].

Muscle metaboreflex activation (MMA) occurs when metabolites produced within active skeletal muscles stimulate chemically sensitive group III/IV afferents, leading to sympathetic excitation [11,12,13]. In addition, changes in the muscle environment including increased temperature and decreased pH might activate accessory receptors and channels that could potentially enhance the MMA response [14,15,16,17,18]. The MMA is sustained after the completion of exercise because metabolites are not removed rapidly [19]. Thus, MMA contributes to increased cardiac sympathetic activity [20,21,22,23,24,25] and could serve as a method to assess the effect of excessive sympathetic output on HRR [19]. For example, a recent study demonstrated that heightened sympathetic output via MMA impairs PHRR [19].

A previous study conducted by Notay et al. [26] examined the effects of acute beetroot juice supplementation on sympathetic nerve activity and suggested that dietary supplements that enhance nitric oxide (NO) bioavailability could potentially reduce efferent sympathetic outflow. Grape seed extract (GSE) is produced through the hydrolysis of specific enzymes which result in highly purified and rich polyphenolic compounds [27]. Several studies have shown that the extract lowers blood pressure by increasing peripheral vasodilation which is linked to the activation of endothelial nitric oxide synthase (eNOS) and the subsequent production of NO [28,29,30]. Although dietary supplementation with GSE promotes NO production, it remains unknown whether the extract improves PHRR in conditions with high sympathetic activation induced by the MMR. Thus, this study hypothesized that sustained sympathoexcitation via MMA slows down PHRR and that GSE supplementation is effective at improving PHRR in young individuals.

## 2. Materials and Methods

A total of 12 healthy males aged between 18 and 30 years were recruited in this study. Individuals with any known cardiovascular or respiratory conditions or conditions that limit physical activity or require antihypertensive medications were excluded from this study. Before any testing began, participants were informed about the potential risks and were required to sign a written consent form. In addition, each participant completed a health screening using a health history questionnaire (HHQ) to confirm that they were in good health. They also received pre-testing guidelines. Participants were asked to avoid smoking, strenuous exercise, and caffeine or alcohol consumption for 24 h prior to each trial. The study was conducted after obtaining an approval from the California Baptist University Institutional Review Board (034-2425-EXP, October 2024).

### 2.1. Experimental Procedures

During the first visit, height (m) and weight (kg) were measured to calculate the body mass index (BMI) using the kg/m^2^ equation. To determine the relative exercise intensity for a workload used in this study, a maximal exercise test was performed using a cycle ergometer (Monark 828, Sweden). The testing protocol began with 1 min of unloaded cycling (warm up) and then the workload increased gradually every minute until the participants could not maintain a pedal cadence of 60 rpm. Pulmonary gases were measured on a breath-by-breath basis using an Ultima CPX metabolic measurement cart (Medgraphic, MGC Diagnostics, Saint Paul, MN, USA). The maximal workload corresponding to the VO2 peak obtained from this test was calculated as the moderate exercise intensity for each participant. To determine the target exercise intensity, the maximal voluntary contraction (MVC) of the dominant forearm was assessed using a handgrip dynamometer. For the MVC test, participants squeezed the dynamometer twice with maximal effort for about 1 s each time, and the higher of the two values recorded was used as their MVC. The static exercise was then performed at 30% of this MVC [31].

During the second visit, participants were seated on a cycle ergometer with their dominant arm positioned on customized armrests adjusted to heart level throughout the test. They completed a cycling exercise at a constant submaximal workload corresponding to 50% of their predetermined maximal workload. Participants sat quietly on a cycle ergometer for 5 min and then performed a submaximal exercise at 60 rpm at moderate exercise intensity (50% VO2 peak) for 6 min. Immediately after exercise, they remained seated on the cycle ergometer quietly for 5 min to measure PHRR.

**Figure 1:**
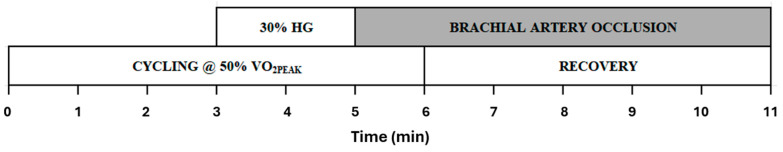
Schematic diagram of experimental design.

Similarly to the protocol used from a previous study [19], an upper limb isometric handgrip exercise and blood flow occlusion were used to minimize the impairment of venous return during exercise in the study. It has been reported that post-exercise circulatory occlusion following isometric handgrip exercise increases sympathetic output without affecting venous return [32]. Prior to the trial, each participant had a familiarization period on how to perform the isometric handgrip exercise at 30% MVC with real-time visual feedback of the force on a display during the submaximal cycling exercise. Immediately 3 min after exercise, they performed the isometric handgrip exercise for 2 min. The blood flow occlusion, using a cuff placed over the brachial artery of a dominant arm (inflation to 200 mmHg), began 5 s before the isometric exercise ended. The occlusion induced MMA and continued for 1 min of cycling exercise and for 5 min of PHRR (Figure 1). The total duration of MMA was 6 min. The same exercise protocol was repeated after either the placebo (PL) or GSE. Participants were instructed to rest, hydrate, and follow an identical diet for 24 h prior to all study days.

### 2.2. Measurement of Hemodynamic Responses

HR, SV, and CO were continuously monitored using a non-invasive impedance cardiography device (Physioflow, Manatec Biomedical, France) both at rest and during the static exercise and post-exercise muscle ischemia (PEMI) phases [28,33]. To measure HR and SV, two electrodes were placed on the left side of the neck, two additional electrodes were placed on the chest for electrocardiography (ECG) measurements, and another two electrodes were placed on the xiphoid process. Blood pressure (systolic and diastolic) was measured from the non-dominant arm at rest and the last 30 s before the PEMI. Mean arterial pressure (MAP) was calculated using the following formula: MAP = [[SBP-DBP] × 1/3] + DBP. CO was determined using the equation CO = SV × HR. Finally, total peripheral resistance (TPR) was calculated as TPR = MAP/CO.

HR data was measured at 5 s intervals throughout the experiment. The PHRR was evaluated between GSE and PL at every min. for 300 s. Heart rate recovery kinetics (Tau) were analyzed using software (SigmaPlot 14, Grafiti LLC, Palo Alto, CA, USA). HR data was analyzed from the end-exercise HR to 5 min after post-exercise recovery. Curve fit was modeled using the following equation: Y = Y0 + A*exp (-b*x). The model parameters were estimated using non-linear regression. Y indicates heart rate at any given time. Y0 + A indicates the starting point of the curve fitting region, and A indicates the amplitude of the change in heart rate from end-exercise to end-recovery. b indicates the time constant (Tau) and the time for heart rate to attain 63% of total amplitude.

### 2.3. Supplementations

Participants were randomly assigned in a double-blind, cross-over design to investigate the effects of acute GSE supplementation on muscle metaboreflex and PHRR kinetics in young individuals. Each participant took either GSE (300 mg, 2 capsules) or a placebo (300 mg, 2 capsules filled with starch) 2 h before their scheduled lab visit. The supplements were made to look identical to prevent any bias from the participants or the testers. There was a washout period of at least 72 h, but no more than a week, between each trial. A washout period of 72 h was selected on the basis of the duration of the effect of GSE observed in our studies [29,34].

### 2.4. Statistical Analysis

All the variables were reported as mean ± standard error (SE). PHRR kinetics parameters, including Tau, amplitude change, end-exercise heart rate, and end-recovery heart rate between the PL and GSE conditions, were analyzed using a paired *t*-test. The one-minute average response for each variable was compared at rest and during exercise. The physiological responses at rest and during submaximal exercise were analyzed using a two-way repeated measures ANOVA (supplementation × conditions). If there was a significant interaction between supplementation and conditions, Tukey’s post hoc test was performed. Statistical significance was set at *p* < 0.05. The sample size was determined through a power analysis, ensuring that the minimum sample size required to detect a statistically significant difference in PHRR before and after supplementation would be met (power = 0.80) [35]. Based on this calculation, 12 participants were required. The effect size (ES) was calculated using Cohen’s d formula which was the mean difference (GSE Vs. placebo) divided by the pooled standard deviation (SD).

## 3. Results

Table 1 shows the physical characteristics of the participants. All participants had a normal resting blood pressure.

Table 2 shows the hemodynamic variables after either supplementation with PL or GSE at rest.

**Figure 2:**
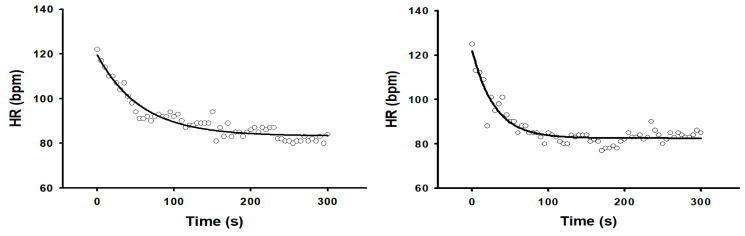
HRR kinetics from a single participant following exercise after supplementation with PL (**left panel**) and GSE (**right panel**).

Figure 1 indicates the experimental design for the current study. Figure 2 shows an example of HRR kinetics from a single participant following exercise after either supplementation with PL or GSE.

**Figure 3:**
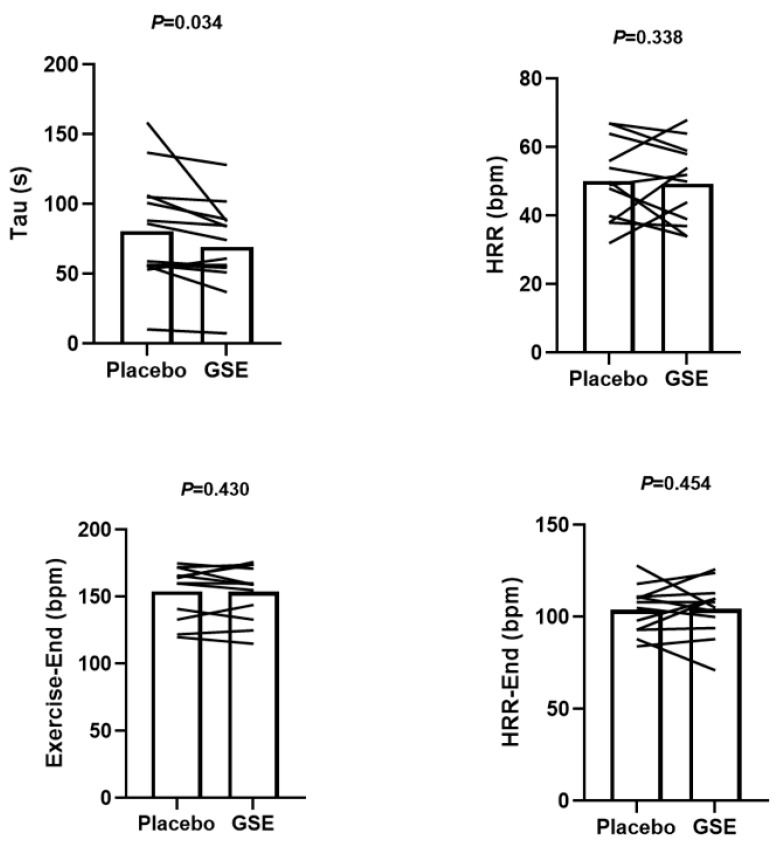
Effect of MMA on heart rate recovery from cycle exercise after supplementation with PL and GSE. Tau indicates heart rate recovery kinetics after supplementation with PL and GSE. Exercise End indicates HR at the end of cycle exercise after supplementation with PL and GSE. HRR indicates the difference between exercise-end HR and end-recovery HR after supplementation with PL and GSE. HRR End indicates end-recovery HR after supplementation with PL and GSE.

Figure 3 shows the effects of MMA on heart rate recovery from the cycle exercise after PL and GSE supplementation. Heart rate recovery kinetics significantly improved following GSE supplementation compared with the PL condition (ES = 0.4). There were no significant differences in HRR, exercise end, and HRR end between PL and GSE supplementation.

**Figure 4:**
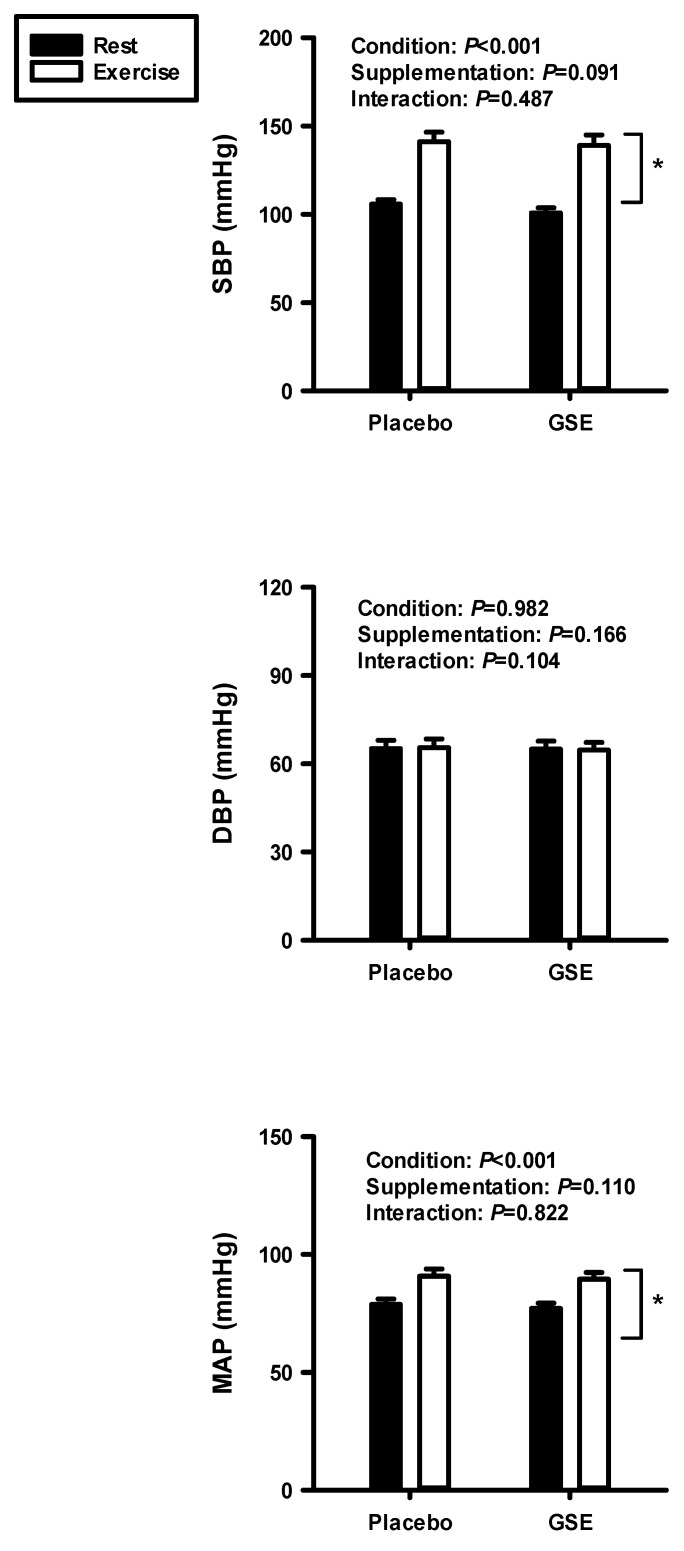
Systolic blood pressure (SBP), diastolic blood pressure (DBP), and mean arterial pressure (MAP) at rest and during cycling exercise after supplementation with PL and GSE. * Vertical bracket indicates significant condition effect.

Figure 4 shows the result of the two-way repeated ANOVA on SBP, DBP, and MAP at rest and during dynamic exercise after supplementation with PL and GSE. There were no significant interactions with SBP, DBP, and MAP between PL and GSE supplementation. However, there were significant condition effects on SBP and MAP.

**Figure 5:**
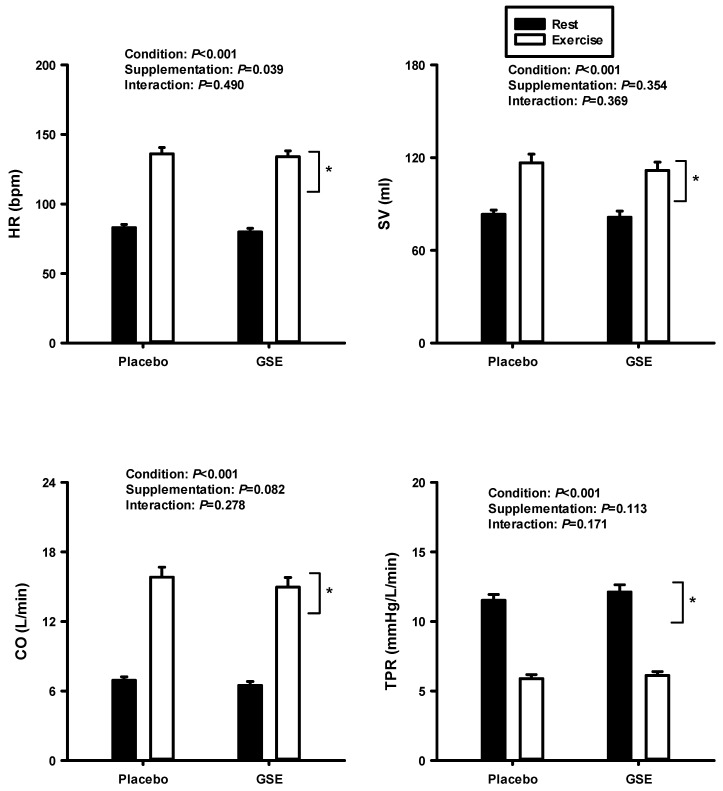
Heart rate (HR), stroke volume (SV), cardiac output (CO), and total peripheral resistance (TPR) at rest and during cycling exercise after supplementation with PL and GSE. * Vertical bracket indicates significant condition effect.

Figure 5 shows the result of the two-way repeated ANOVA on HR, SV, CO, and TPR at rest and during dynamic exercise between PL and GSE supplementation. There were no significant differences in HR, SV, CO, and TPR between the two conditions. However, there were significant condition effects in all variables.

To confirm that the circulatory occlusion following the isometric handgrip exercise activated MMA, MAP was measured with the same trial on cycle ergometry on separate days. Our results indicate that MAP was increased from resting (76.4 ± 2.3) to exercise (81.9 ± 3.1), confirming that MMA increased sympathetic activity.

## 4. Discussion

The main finding in this study was that acute GSE supplementation significantly improved HRR kinetics during enhanced sympathetic activity induced by MMA in young individuals. This result supported the hypothesis that GSE could mitigate sympathoexcitation induced by MMA via an increase in NO production. BP tended to attenuate due to reduced peripheral vasoconstriction following GSE supplementation, but there was no significant difference between the two conditions. Thus, these findings suggest that GSE can act as a dietary ergogenic supplement capable of reducing sympathetic activity and enhancing HRR kinetics.

### 4.1. Heart Rate Recovery Kinetics and MMA

The autonomic control of HR can be evaluated using the assessment of HRR immediately following exercise [36,37]. Slow HRR following exercise is related to all-cause mortality in healthy populations and pathological conditions like hypertension [38,39,40]. Slow HRR accounts for decreased parasympathetic and overactive sympathetic activity [8,19]. HRR is predominantly influenced by parasympathetic reactivation during the initial phase of recovery. However, the role of the sympathetic activity to HRR is not fully elucidated. Along with the findings above, this study is intended to evaluate the effect of dietary supplementation with GSE on HRR after exercise, since GSE is known to increase the production of NO which inhibits sympathetic output [26]. In our study, HRR kinetics significantly improved with GSE supplementation compared with PL. Because of these findings, it seems reasonable that GSE-induced NO production buffers the sympathetic output enhanced via MMA. A previous study demonstrated that augmented sympathetic activation slowed post-exercise HRR in healthy individuals [19]. Taken together, GSE can act as a dietary nutraceutical capable of inhibiting sympathetic activity and improving HRR.

There were no significant differences in HRR, exercise end, or HRR end after PL and GSE supplementation. This study suggests that HRR kinetics could be a good index after exercise to evaluate autonomic function. It may be an important finding to consider when assessing HRR in clinical settings.

### 4.2. Hemodynamic Responses to GSE Supplementation

The results of this study indicated that acute GSE supplementation did not significantly alter resting or post-exercise hemodynamic responses. Although previous studies have suggested that GSE can enhance endothelial nitric oxide (NO) production and reduce BP via peripheral vasodilation in prehypertensive or obese individuals [28,29], no effects were observed. This discrepancy may be due to a younger participant profile who are normotensive.

### 4.3. Limitations

All participants in this study were young and healthy. This limits the applicability of the findings to other populations, such as older adults or individuals with cardiovascular diseases. Previous studies demonstrated that age and cardiovascular health significantly influence cardiac autonomic function and HRR [8,41]. Therefore, the effects of GSE supplementation on HRR should be investigated in populations with varying age groups and those at higher cardiovascular risk.

Although previous research supports the role of GSE in promoting NO production [28,29,30], this study did not directly measure NO levels which limits the ability to establish a direct mechanistic link between GSE supplementation and peripheral vasodilation. Furthermore, sympathetic activity was not measured directly as it can be invasive and require specialized techniques. The direct measurement of sympathetic activity would provide a more comprehensive understanding of the effects of MMA and the mitigating influence of GSE.

## 5. Conclusions

This study shows that acute GSE supplementation significantly improves HRR kinetics during MMA in young individuals. These results support that GSE may improve cardiac autonomic function and accelerate HRR following exercise, potentially reducing the risk of cardiovascular disease. However, further studies are needed to confirm these findings and investigate the long-term effects of GSE on cardiovascular health. In clinical settings, these findings suggest that GSE can have healthy benefits for individuals at a higher risk of cardiovascular disease such as hypertension and heart failure.

## Figures and Tables

**Table 1:** Physical characteristics of participants.

Variables	(*n* = 12)
Age (yrs)	22.2 ± 1
Height (cm)	176.3 ± 2
Weight (kg)	75.3 ± 3
Body mass index (kg/m^2^)	24.3 ± 1
SBP (mmHg)	101.7 ± 3.2
DBP (mmHg)	65.2 ± 2.8
HR (bpm)	68.7 ± 2

Values are expressed as mean ± standard error. SBP: systolic blood pressure; DBP: diastolic blood pressure; HR: heart rate.

**Table 2:** Hemodynamic variables after either supplementation with PL or GSE at rest.

Variables	(*n* = 12)	
	PL	GSE
HR (bpm)	83 ± 2.4	80.0 ± 2.6
SV (mL)	83.4 ± 2.7	81.4 ± 4.0
CO (L/min)	6.9 ± 0.3	6.5 ± 0.3
SBP (mmHg)	105.8 ± 2.5	100.8 ± 3
DBP (mmHg)	65.2 ± 2.8	65.5 ± 2.8
MAP (mmHg)	78.7 ± 2.3	77.3 ± 2.3
TPR (mmHg/L/min)	11.5 ± 0.4	12.4 ± 0.6

Values are expressed as mean ± standard error. HR: heart rate; SV: stroke volume; CO: cardiac output; SBP: systolic blood pressure; DBP: diastolic blood pressure; MAP: mean arterial pressure; TPR: total peripheral resistance.

## Data Availability

The raw data supporting the conclusions of this article would be made available by the authors on request.
